# Schmallenberg virus neutralising antibody responses in sheep

**DOI:** 10.1186/s12917-019-2139-7

**Published:** 2019-11-28

**Authors:** Scott Jones, Laura Eden, Heather McKay, Nicola Bollard, Stephen Dunham, Peers Davies, Rachael Tarlinton

**Affiliations:** 1 School of Veterinary Medicine and Science, University of Nottingham, Sutton Bonington Campus, Leicestershire, LE12 5RD UK; 2 Department of Epidemiology and Population Health, University of Liverpool, Liverpool, UK

**Keywords:** Schmallenberg virus, Immunity, Antibody responses, Virus-neutralisation test, Quantitative PCR, Sheep

## Abstract

**Background:**

Schmallenberg virus (SBV) is a midge borne virus of cattle and sheep. Infection is typically asymptomatic in adult sheep but fetal infection during pregnancy can result in abortion, stillbirth, neurological disorders and malformations of variable severity in newborn animals. It was first identified in Germany and the Netherlands in 2011 and then circulated throughout Europe in 2012 and 2013. Circulation in subsequent years was low or non-existent until summer and autumn 2016, leading to an increased incidence of deformed newborn lambs and calves in 2016–17. This study reports SBV circulation in October 2016 within a group of 24 ewes and 13 rams. The ewes were monitored at 3 times points over an 11 week period (September to December 2016).

**Results:**

Most ewes displayed an increase in SBV VNT with antibody titre increases greater in older, previously exposed ewes. Two ewes had SBV RNA detectable by RT-qPCR, one on 30/09/16 and one on 04/11/16. Of these ewes, one had detectable serum SBV RNA (indicating viraemia) despite pre-existing antibody. The rams had been previously vaccinated with a commercial inactivated SBV vaccine, they showed minimal neutralising antibody titres against SBV 8 months post-vaccination and all displayed increased titre in October 2016.

**Conclusion:**

This data suggests that SBV circulated for a minimum period of 5 weeks in September to October 2016 in central England. Ewes previously exposed to virus showed an enhanced antibody response compared to naïve animals. Pre-existing antibody titre did not prevent re-infection in at least one animal, implying immunity to SBV upon natural exposure may not be life-long. In addition, data suggests that immunity provided by killed adjuvanted SBV vaccines only provides short term protection (< 8 months) from virus.

## Background

Schmallenberg Virus (SBV), an *Orthobunyavirus* of the *Bunyaviridae* family is a recently emerged virus first identified within cattle in Germany and the Netherlands during the summer and autumn of 2011, following which SBV was associated with deformities seen in newborn calves and lambs [1, 2]. Infection in adult cattle results in mild disease with clinical signs including pyrexia, decreased milk production and diarrhoea while it is typically asymptomatic in adult sheep [3, 4]. Of much greater economic importance is the occurrence of fetal infection which can result in abortion, stillbirth, neurologic disorders and limb malformations in newborn animals with variable severity [5]. This range of clinical signs has been suggested to be the result of infection at different gestational stages with early infection causing most severe cases, similar to Akabane virus, another *Orthobunyavirus* [6].

In common with other *Orthobunyaviruses*, SBV is a vector borne virus spread by *Culicoides sp.* biting midges; with midges of the *Culicoides obsoletus* complex the main vectors in Europe [7, 8]. These species have a host range extending over much of Europe. The midge’s lifecycle is heavily temperature dependent with peaks in numbers of midges occurring in late summer and very little activity in winter, with overwintering in livestock housing a major method of survival in colder periods of the year [9, 10].

Circulation of SBV in Europe continued during 2012 and 2013 with reported cases in 13,846 holdings including alpacas, bison, cattle, sheep, goats, deer, buffalo and moose from 29 European countries [11]. Within the United Kingdom (UK) seroprevalences of up to 73% were reported in the worst affected counties [12]. Following these outbreaks, three commercial SBV vaccines were made available. These vaccines, based on inactivated, adjuvanted virus proved to be effective in prevention of SBV associated disease upon implementation in cattle and sheep [13, 14]. In subsequent years few clinical cases of SBV disease were reported, presumably due to very high seroconversion rates nationally and the resulting herd immunity to re-infection [15]. Subsequent vaccine uptake was low due to perceived low risk of infection, with fewer than 14% of sheep holdings in some regions using it. Thus resulting in a cease in production of vaccines until recently, when the Zulvac SBV vaccine (Zoetis UK Limited, Surrey, UK) was reintroduced to the commercial market [16].

However low levels of virus circulation in 2014–16 (presumably due to the high numbers of susceptible hosts which seroconverted in the initial outbreak) meant that animals born in that time frame (a substantial portion of the 2016 UK sheep flock) were naive to the virus and vulnerable to infection [17]. Recently, SBV was identified in a large number of animals in the UK, Ireland and Belgium in late summer/autumn of 2016, confirmed by both seroconversion and the identification of SBV RNA positive *Culicoides sp* with the subsequent appearance in the 2016–17 lambing season of large numbers of deformed fetuses [18]. It is of particular importance to note that this time period (August to September) coincides with the breeding season of sheep (August to December) in the majority of European production systems.

This study reports an accurate timing of transmission in a UK sheep flock participating in an artificial insemination trial. It reports antibody responses and RT-qPCR virus detection upon natural re-exposure to SBV in two groups of animals. Thirteen rams that had previously received one of the commercial vaccines in June 2014 and 24 ewes from a flock known to have had natural virus infection in March–April 2013 during the initial outbreak of the virus [19]. This flock had never been vaccinated. The 15 older ewes (born 2010–13) had been previously naturally exposed to the virus, the 9 younger ewes born in 2014–15, a period of low or no viral circulation in southern England where this flock was located, were presumed naive to the virus [16].

## Results

**Fig. 1:**
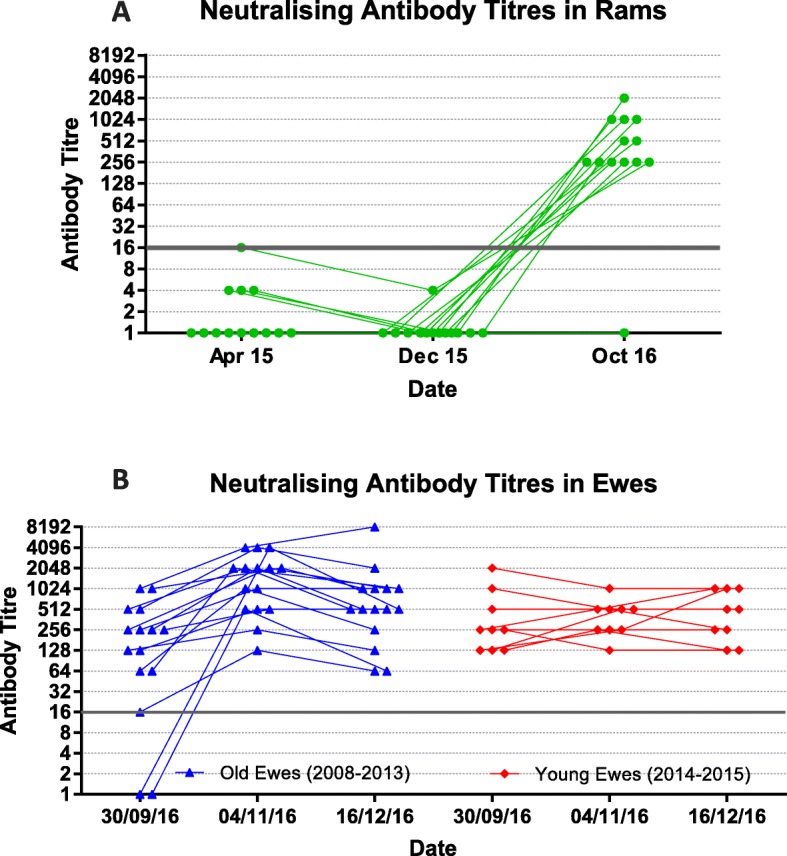
Neutralising Antibody Titres for Ewes and Rams over Three Time Points During/Prior to SBV Circulation. Antibody titres from three time points **a** Apr 2015, Dec 2015 and Oct 2016 from 13 rams previously vaccinated against SBV and **b** 30th Sept 2016 (week 0), 4th Nov 2016 (week 5) and 16th Dec 2016 (week 11) from 15 ewes born between the years 2008 and 2013 (blue) and 9 ewes born between 2014 and 2015 (red). Grey line signifies the minimum detection level for antibodies (16) using this assay

The rams (7 Abermax and 6 Aberfield) were vaccinated according to manufacturer’s instructions with a single dose of Bovilis SBV (MSD animal health) in June 2014 by the breeder, at 14 months of age. Only one of the thirteen rams had a virus neutralising antibody titre (16) at the assay minimum detection level for sheep upon blood sampling in April 2015 at 8 months post vaccination, this titre dropped to 4 by December 2015 at 18 months post vaccination (Fig. 1a).

The 24 Exlana ewes were divided into two subgroups, those born between 2010 and 2013 (*N* = 15) and therefore alive during the previous SBV outbreak and those born after, between 2014 and 2015 (*N* = 9). This flock of ewes had not been vaccinated for SBV. Of 24 ewes, 2 did not show detectable neutralising antibodies (VNT ≥ 16) at all time points, 3064 and 3253. These two ewes, from the older group both showed negative titres at the initial time point (30/09/16) (Fig. 1b). Nineteen of the 24 displayed a rising antibody titre over two subsequent time points at some stage in the study, the 5 remaining ewes were all younger ewes born in 2014 and 2015. Thirteen ewes displayed a 4 fold or greater increase in antibody titre in paired samples 4 weeks apart, generally accepted as an indication of recent exposure to a pathogen. The median antibody titres for both older and younger ewes were the same at the beginning and end time points of the study (256 and 512, respectively).

At week 0 and 11 (30/09/16 and 16/12/16, respectively), titres between groups were indistinguishable ranging from 0 to 1024 with the exception of one ewe (1133) with a titre of 8192 during week 11. At week 5 (04/11/16), ewes born between 2010 and 2013 showed a larger neutralising antibody response following exposure to SBV when compared to younger animals. Comparison of the increase in antibody titres at 04/11/16 (middle time point) between older and younger ewes by regression model found a significantly greater rise in titre of older ewe.

**Fig. 2:**
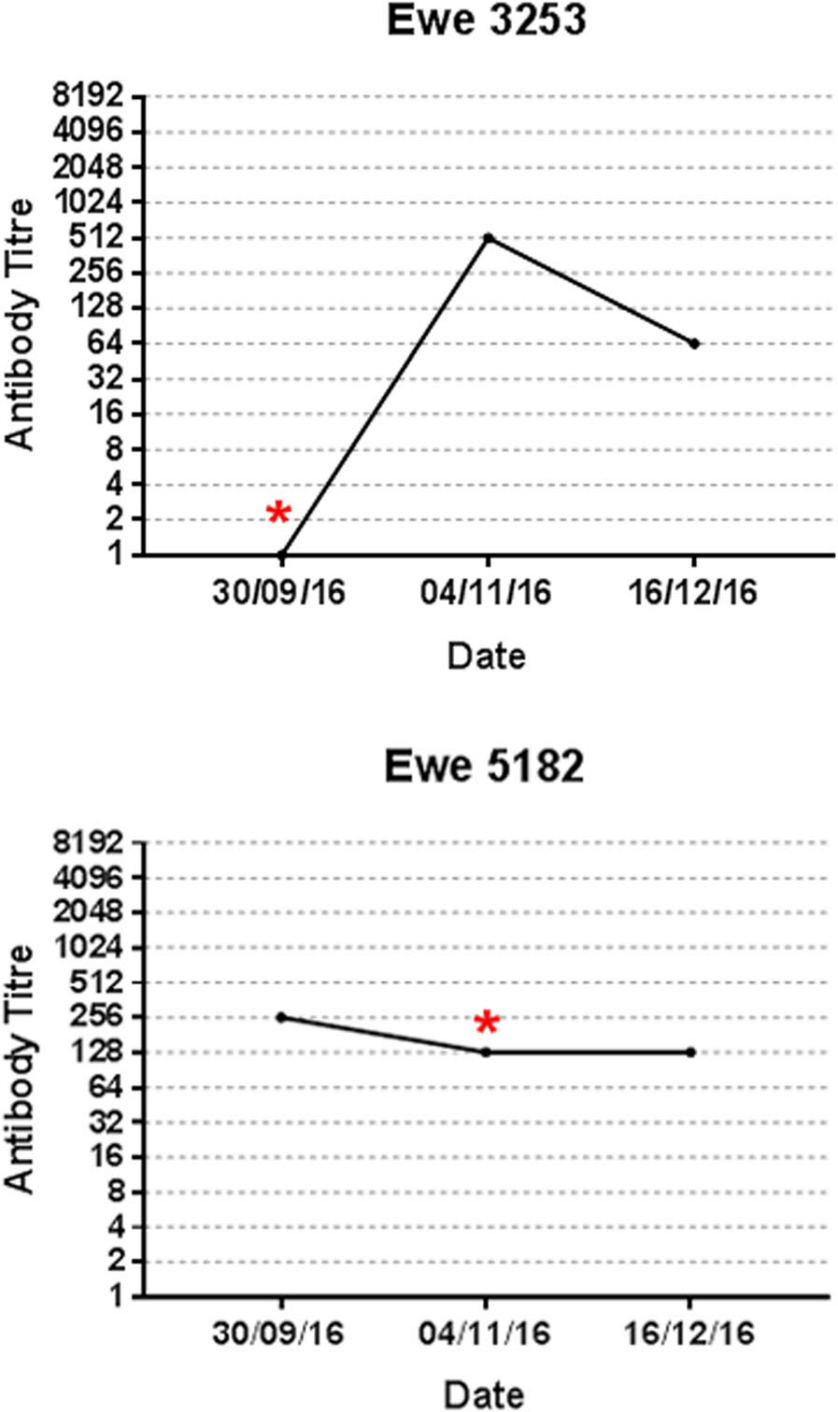
Neutralising Antibody Titres of Two Ewes over Three Time Points for SBV by RT-qPCR. Antibody titres of two ewes over three time points (week 0, 5 and 11) commencing 29th Sept 2016. Red asterisk indicates presence of SBV nucleic acid, determined by RT-qPCR for SBV S fragment

RT-qPCR was carried out on sera from the ewes at each time point to detect viraemia. One animal at each time points, 0 and 5 weeks, were RT-qPCR positive for SBV viraemia (ewes 3253, an old animal born in 2013 with a CT value of 30.87, and 5182, a young animal born in 2015, with a CT value of 30.20, respectively) (Fig. 2).

## Discussion

The presence of virus neutralising antibodies within only one of thirteen rams 14 months post vaccination suggest that the protection provided by the Bovilis SBV vaccine (MSD animal health) is indeed short lived and that animals would require booster doses before annual breeding. This is shorter than the estimated 12 months assumed following the initial release of the vaccine [20]. Though, as the purpose of the vaccine was specifically to prevent foetal infection, short term immunisation could provide protection for animals during pregnancy assuming vaccination occurred at least 3 weeks prior to breeding, with subsequent yearly boosters [21]. We cannot categorically rule out the possibility of natural exposure to SBV in these rams, though other studies have shown that duration of immunity in sheep recovered from wild type virus infection sheep is long lasting (> 16 months) and have indicated that circulation of SBV in the UK and the Republic of Ireland was at very low levels in 2013–14 and practically non-existent in 2014–15 [16, 22, 23]. We also cannot exclude the possibility that the vaccine was not administered correctly, was compromised due to improper storage or specific batch issues. However, detectable titres in at least one individual would indicate this was unlikely. Although, it is important to note that indications of infection does not translate to indications of disease, as vaccine may be protective in preventing clinical disease. Further work is required in determining the clinical presentation of SBV infection in vaccinated animals following re-infection.

The lack of change in SBV neutralising titres between February and December 2015 is consistent with other UK studies indicating that SBV circulation was not detectable in the UK sheep population in 2015 [24]. In comparison, the final blood samples taken from these rams collected in October 2016 showed increases in antibody titres suggestive of recent exposure therefore inferring SBV circulation during this time (Fig. 1a). As the rams were sacrificed at the third blood sampling time point further blood samples and therefore demonstration of a 4 fold increase in antibody titre were not able to be performed. In comparison, antibody titres within 13 of 24 ewes showed a 4 fold increase, generally accepted as an indication of recent exposure to a pathogen.

The larger neutralising antibody responses seen at week 5 (04/11/16) in ewes born between 2010 and 2013 when compared to younger animals, found to be significant by regression modelling is suggestive of an anamnestic response on re-exposure to virus in the older animals, likely due to previous infection.

The period of viraemia for SBV in sheep is short with viral RNA detectable starting from days 1 to 6 post infection for a duration of 4 to 5 days and may overlap with the development of antibody [13, 23, 25]. The existence of antibody 4 weeks prior to the detection of viraemia in animal 5182 would indicate that in contrast to the previously published experimental studies in sheep pre-existing VNT for SBV does not always protect against natural infection [23]. This discrepancy may be due to difference in infective dose of virus (uncontrolled in natural exposure) or differences in virus strain or sheep responses (perhaps due to breed differences).

Taken together (seroconversion and viraemia) these data indicate that SBV was circulating in central England between 29th September 2016 and 27th October 2016. This is consistent with contemporaneous data from surveillance monitoring of deformed foetuses with circulation extrapolated from birth dates of affected lambs and calves in Scotland between October 2016 and January 2017 [26], and in England and Wales (http://ahvla.defra.gov.uk/vet-gateway/schmallenberg/index.htm) between August and December 2017. Of note this is later than the peak in predicted midge abundance (August to September) in models of midge number based on historical data sets [9], though with increasingly warm autumns likely in the future as a part of climate change this pattern of late autumn viral circulation of midge borne diseases is likely to persist. This elongation in circulation past the predicted period places circulation well within the sheep breeding season (and vulnerable period of gestation for lambs) in the UK.

The serological data presented here are in contrast to that in the limited number of previous studies of natural and vaccine induced immunity in sheep. Previous research has found that following experimental infection, animals developed protective immunity that was maintained for at least 16 months, while similar studies in naturally infected cattle showed protective immunity for at least 24 months [27, 28]. The ewes in this study were sourced from a flock known to have been exposed to SBV in winter/spring 2013 (Davies and Daly 2015), and were known not to have been vaccinated. Some of them had detectable antibody titres in September 2016 (42 months later). From this data we could not identify whether this immunity was protective but previous experimental studies have not shown an antibody boost response such as that demonstrated here in animals with protective immunity [13, 14, 23]. It seems likely that these animals did not gain lifelong protection from SBV infection from natural infection. It is possible that annual re-exposure to the virus is necessary to maintain protective immunity, making such long periods of undetectable virus circulation, as occurred between 2013 and 2016, a significant problem with the future management of SBV outbreaks in Europe. Management is further complicated with the unpredictable nature of the supply of SBV vaccines.

## Conclusion

The data presented here provide an accurate circulation date for SBV in central England over a period between September 29th and October 27th 2016. This correlates well with the predicted peak viral circulation extrapolated from numbers of deformed lambs and calves submitted for diagnostics in the following spring. This adds further evidence based assessment to the management recommendation to shift timing of mating until October to avoid SBV risk. This study indicates that immunity against SBV following vaccination with a whole virus inactivated adjuvanted vaccine does not provide long term protection against the virus and that booster vaccinations annually before breeding would be necessary in sheep if this vaccination strategy is followed. More seriously the study indicates that sheep previously naturally exposed to the virus may not be fully protected against it on re-exposure after a substantial time lapse in virus exposure. Previously exposed animals do however display an exaggerated antibody response on re-exposure when compared with naive animals indicative of some immunological memory. The current study is observational and therefore cannot definitively answer questions about protective long term immune responses following natural exposure, nonetheless it does provide additional data that can be used for sheep management in a complicated epidemiological situation.

## Methods

Blood was taken from 13 rams (7 Abermax and 6 Aberfield) and 24 ewes (Exlana) participating in an artificial insemination trial over an 18 month and 11 week period, respectively. Animals were purchased directly from commercial flocks with known disease status for SBV. Animals were kept in field conditions at pasture with shelter. The land holder where this study was performed, the University of Nottingham, granted permission through the university’s ethics process. All procedures carried out were approved by the UK Home Office under the ‘Animals (Scientific Procedures) Act 1986’ (licence no. PPL 30/3367). Following completion of the insemination trial animals were humanely euthanised by captive bolt.

Virus neutralisation tests (VNT) were performed as previously reported [29]. Sera for each animal at each time point were repeated in duplicate. Regression modelling of results was carried out using MLwiN multilevel modelling software.

Extraction of RNA from blood sera was carried out by use of the QIAamp viral RNA Mini Kit (Qiagen, Manchester, UK) following the manufacturers recommended protocol. RNA was eluted with 30 μl nuclease free water and 1 μl used for synthesis of cDNA using the moloney murine leukemia virus (M-MLV) reverse transcriptase (RT) (Promega, Southampton, UK) according to manufacturer’s protocol.

Identification and quantification of viral loads within cDNA from blood sera at time of sampling was carried out by quantitative polymerase chain reaction (qPCR) as previously reported [30]. Reactions were performed using the LightCycler® 480 system (Roche, Pleasanton, USA). Subsequent analysis was carried out using LightCycler® 480 software.

## Data Availability

Serum samples from this study may be obtained on request from the corresponding author. The datasets used and/or analysed during the current study are available from the corresponding author on request.

## Abbreviations

DMEM: Dulbecco’s Modified Eagle Medium
FCS: Foetal calf serum
M-MLV: Moloney murine leukemia virus
qPCR: Quantitative polymerase chain reaction
RT: Reverse transcriptase
S Fragment: Small fragment
SBV: Schmallenberg virus
UK: United Kingdom
